# 510 5-Year Retrospective Analysis of a Vented Early Mobility Algorithm in the Burn ICU

**DOI:** 10.1093/jbcr/irac012.141

**Published:** 2022-03-23

**Authors:** Audrey M ONeil, Cassandra Rush, Laura Griffard, David Roggy, Brett C Hartman, Allison N Boyd

**Affiliations:** Eskenazi Health Hospital, Indianapolis, Indiana; Eskenazi Health, Indianapolis, Indiana; Eskenazi Health, Pendletion, Indiana; Richard M Fairbanks Burn Center, Indianapolis, Indiana; Richard M. Fairbanks Burn Center, Indianapolis, Indiana; Eskenazi Health, Indianapolis, Indiana

## Abstract

**Introduction:**

The practice of early mobilization on mechanically ventilated patients in ICU settings has received significant attention within recent literature, however limited research to date has focused specifically on the burn population. The purpose of this single center retrospective analysis was to review the use of a burn critical care mobility algorithm, to determine the safety and feasibility of early mobility programs in the burn population, expose limitations preventing mobility progression at our facility, and discuss unique challenges to early mobility with burn patients.

**Methods:**

A retrospective review was completed for all intubated burn center admissions between January 2015 to December 2019. Burn Therapy notes were then reviewed for all intubated patients from initial evaluation until each patient was either extubated or underwent a tracheostomy. Data was collected using the stages of the mobility algorithm.

**Results:**

In the 5 years following initial implementation, the vented mobility algorithm was utilized on 127 patients with an average TBSA of 22.8%. Of these patients, 25 were transitioned to comfort care measures due to the extent of their burn injuries but were included in the review. The average intubated days were 8.05 days and the average length of stay was 26 days. No adverse events occurred during treatment with the algorithm. Stage 1: PROM/AROM was completed with 100% of patients (n=127). Chair mode of bed, stage 2a, was utilized in 39.4%(n=50) of patients, while 15.8% (n=20) of patients were dependently transferred to the cardiac chair in stage 2b. Stage 3 (sitting on the edge-of-bed) was completed with 25% (n=32) of patients, with only 11% (n=14) progressing to stage 5 (standing), and 3.9% (n=5) actively transferring to a chair. In 5 years, only 4.7% (n=6) reached stage 6 (ambulation). The most common treatment limitations were medical complications (33%) including: unstable medical status, agitation, sedation, cultured epithelial autograft placement, and orthopedic restrictions. Line placement, including femoral and pedal catheters , was also found to be a barrier to the use of our algorithm in 21% of held patient treatments.

**Conclusions:**

Retrospective analysis demonstrates that early mobilization during mechanical ventilation is safe and feasible with in the burn population despite challenges including airway stability, sedation, and line limitations. Dedicated burn therapy staff and team coordination is required to optimize management during the critical care stage of recovery and improve outcomes for survivors.

